# Advances of Single‐Atomic Cobalt Catalysts in Liquid‐Phase Selective Oxidative Reactions

**DOI:** 10.1002/smsc.202300079

**Published:** 2023-10-26

**Authors:** Jiaquan Li, Kai Wang, Yijiao Jiang, Jun Huang, Shaomin Liu

**Affiliations:** ^1^ School of Chemical and Biomolecular Engineering Sydney Nano Institute The University of Sydney Sydney NSW 2037 Australia; ^2^ WA School of Mines: Minerals, Energy and Chemical Engineering Curtin University Perth WA 6102 Australia; ^3^ Department of Engineering Macquarie University Sydney NSW 2109 Australia

**Keywords:** cobalt, oxidants, reaction mechanism, selective oxidation, single-atom catalysts

## Abstract

Single‐atom catalysts (SACs) composed of atomically dispersed metal‐active sites embedded in supporting substrates are attracting increasing attention in liquid‐phase selective oxidation reactions with joint merits of both advanced catalytic efficiency and high stability. Co‐based SACs present superior performance in several model oxidative reactions against many other metals, thus they are recognized as a promising solution to the current high‐cost noble‐metal catalysts required for the synthesis of fine chemicals. In this review, the up‐to‐date research on the synthesized Co–SACs in selective oxidation applications is summarized. The strategies of the preparation of Co–SACs with diverse Co‐loading levels and well‐tuned morphologies and chemical structures are showcased, as well as the characterization techniques of the SACs. The applications of Co–SACs in a series of selective oxidation reactions and the influence of different oxidants on the overall reaction efficiency are discussed. In addition, the progress of the mechanism exploration involving active‐site identification, catalytic activation of oxidants, and oxidation pathway elucidation is highlighted. Meanwhile, the existing challenges and the future efforts for the development of the Co–SAC reaction system in selective oxidation processes are outlined.

## Graphical abstract

Earth‐abundant cobalt‐based single‐atom catalysts (Co‐SACs) are promising for selective oxidation reactions to replace the traditional high‐cost noble‐metal catalysts. In this review, the current application of Co‐SACs in liquid‐phase selective oxidations regarding the synthesis methods, the types of reactions, various oxidants, the mechanisms for oxidants activation and organic oxidation, and the challenges faced with the current research is showcased.

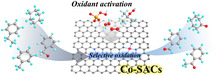

## 1 Introduction

The liquid‐phase selective oxidations of raw and cheap organics into valuable functionalized products are vital in the production of many chemicals and intermediates in synthetic chemistry.^[^ ^1^ ^]^ Typical reactions include the conversion of aromatic/aliphatic alkanes and alcohols into aldehydes, ketones, or acids, which are extensively applied as building blocks in industries of fine chemicals, pharmaceuticals, agricultural chemicals, etc. In the scenario of catalysis, both noble‐metal‐ and transition‐metal‐based homogeneous catalysts such as Pd, Ru, Ni, Cu, and Co^[^ ^2^ ^]^ play an important part in the current chemical industry owing to the high catalytic activity. The reactions between homogeneous catalysts and reactants occur in the same phase to achieve the maximum atomic utilization and catalytic efficiency, whereas the harsh conditions, additional product separation, and catalyst recycling remain the critical issues. In this context, attention was focused on heterogeneous‐metal‐based catalysts for economic and environment‐favorable oxidation processes. Noble metals Pt, Pd, Au, and Ru and transition metals Fe, Co, Cu, and Cr were frequently used to form supported nanoparticle complexes to conduct the catalytic oxidations.^[^ ^3^ ^]^ However, the heterogeneous reaction occurs on the surface of the catalysts, thus the metal utilization efficiency was limited by the metal sites confined inside the particles. Therefore, it poses a significant challenge to achieve improved dispersion of metal‐active sites on heterogeneous catalysts. Moreover, there is a growing demand of sustainability in today's chemical industry, but the current fine‐chemical synthesis still heavily relies on the highly corrosive and toxic reagent to attain high productivity. To substitute the traditional chemical synthesis routes, novel catalysts with enhanced atomic efficiency and stability to activate green oxidants for selective oxidation reaction are highly desired.

The emergence of single‐atom catalyst (SAC) shed light on bridging the homogeneous and heterogeneous catalysis processes.^[^ ^4^ ^]^ First proposed by Zhang and co‐workers in 2011, the concept of SAC refers to atomically isolated metals deposited on supporting materials such as metal oxides, molecular sieve, and metal‐free carbon materials.^[^ ^5^ ^]^ Compared with the common heterogeneous metal catalysts, SACs contain uniformly dispersed active sites of single‐metal atoms with the potentially maximized atomic efficiency.^[^ ^6^ ^]^ Nevertheless, the thermodynamic surface free energy of the metal species dramatically increases when they are separated as single atoms, leading to a strong tendency of aggregation of single atoms into nanoparticles during the formation of SACs. The fabrication of SAC with high loading of single‐metal atoms still remains a challenging task. The past decade witnessed the rapid growth of research enthusiasm in SAC, with the development of various synthesis strategies and a wide range of application fields including thermal catalysis, photocatalysis, and electrocatalysis.^[^ ^7^ ^]^ Specifically, SAC exhibits a great potential in liquid‐phase selective oxidation processes, attributed to its tunable and concise metal‐active sites, advanced selectivity, high resistance against heat/acidic environment, synergistic effect with supporting materials, and unique reaction pathways.^[^ ^8^ ^]^ The most frequently reported single atom (SA) metals for liquid‐phase oxidation reactions include Pd, Au, Rh, Ru, Pt, Fe, Co, Cu, and Ni.[^8^, ^9^] Noble metals generally exhibit superior catalytic activity than other catalysts, while the high cost and scarcity of noble metals drive more and more research turning to earth‐abundant transition metals, especially Co‐ and Fe‐based SACs. Co‐based catalysts presented great potential in the activation of many oxidants owing to its less negative reduction potential compared with many other transition metals. For instance, it was found that Co outperformed the other transition metals including Mn, Ni, and Fe in the activation of hydrogen peroxide for the selective oxidation of different alcohols.^[^ ^10^ ^]^ Above that, Co SAC supported on N‐doped carbon was reported to deliver higher activity than Fe‐, Ni‐ and Cu‐based SACs in the oxidative esterification of alcohols.^[^ ^11^ ^]^ Considering the superior catalytic performance of Co–SACs in selective oxidation reaction system, it's important to fundamentally discuss their current development and application status, and the challenges and direction for further improvement. Therefore, the up‐to‐date progress of Co–SACs in liquid‐phase oxidative organic synthesis processes is summarized herein for an in‐depth understanding. The synthesis of Co–SACs, the reaction systems of their applications, the employed oxidants, and the proposed mechanisms from the reported work are compared to derive some correlations between catalyst structure and activity performance. The future efforts hopefully to upgrade the oxidation process over Co–SACs are also suggested.

## 2 Synthesis and Characterization of Co‐Based SACs

### 2.1 Influence of the Preparation Methods on the Synthesis of Co–SACs

**Figure 1:**
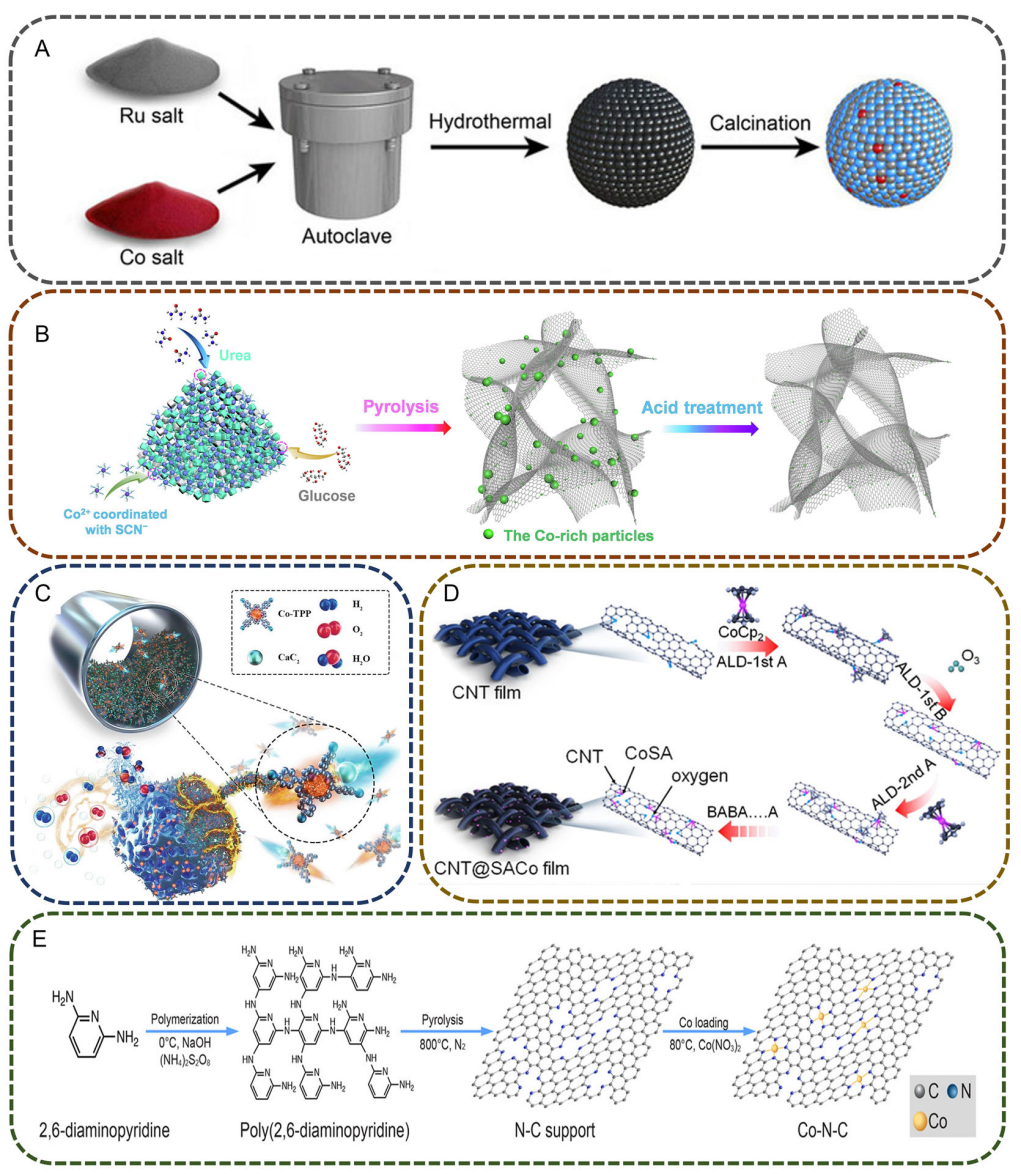
Schematic illustration of the representative synthesis process of cobalt‐based single‐metal catalysts (Co–SACs). A) Synthesis of single atom Co on ruthenium oxide by hydrothermal precipitation. Reproduced with permission.^[^ ^12^ ^]^ Copyright 2021, Wiley‐VCH GmbH. B) Preparation of single atom Co by pyrolysis method. Reproduced with permission.^[^ ^19^ ^]^ Copyright 2019, Elsevier. C) Ball milling process for Co single atom catalyst preparation. Reproduced with permission.^[^ ^20^ ^]^ Copyright 2021, Elsevier. D) Atomic layer deposition treatment to produce carbon nanotube‐supported Co atoms. Reproduced with permission.^[^ ^21^ ^]^ Copyright 2020, The American Chemical Society. E) Schematic of the synthesis of polymer support and impregnation of Co atoms. Reproduced with permission.^[^ ^22^ ^]^ Copyright 2020, The American Chemical Society.

Fundamentally, the preparation methods of SACs fall into two categories, namely the in situ generation and post‐preparation. The in situ fabrication of SACs includes typical methods such as hydrothermal precipitation,^[^ ^12^ ^]^ pyrolysis,^[^ ^11^, ^13^ ^]^ and ball milling,^[^ ^14^ ^]^ while the post‐preparation pathways mainly involve atomic layer deposition (ALD),^[^ ^15^ ^]^ wet impregnation,[^9^, ^16^] adsorption,^[^ ^17^ ^]^ and photochemical reduction.^[^ ^18^ ^]^ These protocols were commonly used in the synthesis of Co‐based SACs for the research of organic selective oxidation reactions. Five representative examples of the synthesis process for Co–SACs from different methods are displayed in **Figure** **1**. Hydrothermal synthesis is used to simultaneously generate supports and highly dispersed single‐atom metal species from the precipitation of the resolved precursors. As shown in Figure 1A, Shah et al. fabricated atomically dispersed Co on the surface of RuO_2_ by one‐pot hydrothermal treatment of ruthenium chloride and cobalt chloride solution in the presence of urea and sodium dodecylbenzene sulfonate at 100 °C.^[^ ^12^ ^]^ The precipitate was further calcined at 400 °C to obtain the final Co–SAC/RuO_2_ with 1.82 wt% Co loading. This method is facile and easy to scale up the production of Co–SACs on various supporting materials such as metal oxides, zeolites, and metallic organic frameworks (MOFs).

Pyrolysis of cobalt and carbon precursors is another in situ method to prepare Co single atoms embedded on carbon‐based materials. For instance, a mixture of urea, glucose, CoCl_2_, and NaSCN in ethanol solution containing C, N, and Co sources was used as precursor of Co–SACs.^[^ ^19^ ^]^ After evaporating ethanol, the remaining solid was annealed at 800 °C for the carbonization of the C source into graphene‐like carbon. The resulting solid contains both Co particles and single‐atom sites, with the former ones removed by acid etching and the Co single atoms were stabilized on the graphene sheets with relatively high concentration of 2.16 wt% (Figure 1B). The pyrolysis method has been extensively used to prepare Co–SACs on carbon materials with high Co atom loading and tunable heteroatom compositions by well‐designed synthesis process.

Figure 1C illustrates the ball‐milling process for Co–SAC production reported by Jin et al.^[^ ^20^ ^]^ Ball milling the mixture of cobalt (II) 5,10,15,20‐tetrakis‐(4′‐bromophenyl) porphyrin (Co–TPP‐Br) and calcium carbide could reconstruct the configuration of the precursors and form new bonds of the substrate. The resultant solid was washed with hydrochloric acid to eliminate the metal particles and impurities and obtain single‐atom Co supported on N‐doped carbon. The whole process is very simple and free of solvent or any other sacrificial components and is suitable for large‐scale production of Co–SACs.

Post‐synthetic method refers to the deposition of Co atoms on pre‐synthesized supports. One of the post‐preparation methods is ALD. As shown in Figure 1D, the carbon nanotube (CNT) support was pretreated with acid etching and oxygen plasma to create abundant oxygen‐functional groups to anchor the Co atom.^[^ ^21^ ^]^ Then, the CNTs were treated with ALD cycles in exposure of (cyclopentadienyl) cobalt and oxygen pulses, respectively. The ALD method can synthesize Co–SACs with well‐controlled thickness of layer growth of atomic and excellent conformity but suffers from mass material waste and long‐time‐deposition treatment.

Figure 1E presents the synthesis of SACo fixed in a polyaromatic macrostructure via impregnation method.^[^ ^22^ ^]^ First, the supporting material for SACo was synthesized by polymerization of 2,6‐diaminopyridine assisted by NaOH and ammonium persulfate in water. The polymer was subsequently annealed at 800 °C. The single‐atom Co was introduced to the polymer by wet impregnation with Co(NO_3_)_2_. The key to the wet impregnation or adsorption method is to establish coordination between Co species and the supporting materials. However, the Co single‐atom content is usually ultralow (<0.1 wt%) to avoid the aggregation of the Co species which limits the catalytic efficiency.

Although various supports have been applied to embed the Co single atoms, such as metal oxides, zeolites, and MOFs, when narrowing down to the Co–SACs employed in liquid‐phase selective oxidation reactions, most of the Co–SACs employed carbon‐based materials, including graphene, CNTs, carbon nitride, and MOF‐derived carbon. The highly favorable selection of carbon support for Co–SAC was due to 1) their merits of high specific surface area from porous or nanostructure and the carbon skeleton ready for heteroatom doping for generating abundant defect sites to immobilize single atoms;^[^ ^23^ ^]^ 2) carbon materials alone can serve as catalysts in liquid‐phase reactions which will enhance the overall catalytic performance of Co–SACs;^[^ ^24^ ^]^ 3) there is synergistic catalytic effect between Co atoms and the supporting carbon materials owing to the extraordinary electrochemical properties of the carbon framework to facilitate the redox cycles in the oxidation reaction; 4) carbon materials are sustainable and cheap and are relatively stable in acidic/basic liquid reactions. In addition, nitrogen is easily doped in carbon matrix as a nonmetallic heteroatom, and Co atoms are readily associated with N species due to the affinity between Co and the lone electron pairs of N atoms, making the Co–N_*x*_ configuration suitable to anchor separated Co atoms through stable chemical bonds.^[^ ^25^ ^]^

**Figure 2:**
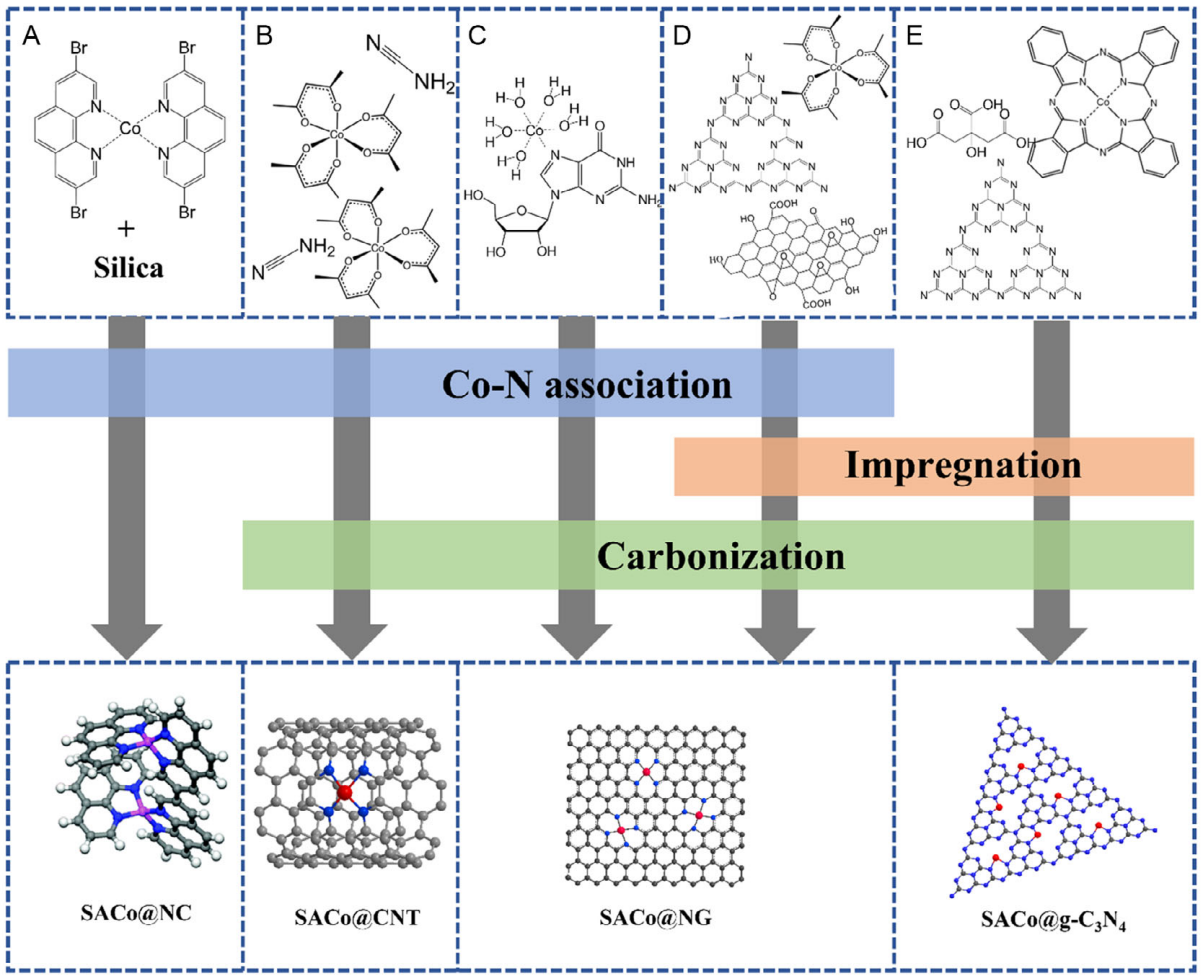
Examples of chemical association of different Co, N, and C precursors in the synthesis of Co–SACs on carbon supports for liquid‐phase selective oxidations. A) Coordination of Co ions with 3,8‐diBr‐Phen for the synthesis of polymer‐supported Co atoms. B) Synthesis of Co‐SAC on carbon nanotubes by cobalt (III) acetyl‐acetonate and cyanamide. C,D) Synthesis of N‐graphene‐supported Co‐SACs. E) Deposition of Co single atoms on carbon nitride from cobalt (II) phthalocyanine. Reproduced with permission.^[^ ^30^ ^]^ Copyright 2019, The Royal Society of Chemistry.

In currently reported work of liquid‐phase selective oxidations, the Co–SACs on carbon supports were mainly prepared by wet impregnation or pyrolysis method. For example, SACo@graphene was produced by adopting commercial graphene oxide and cobalt nitrate/dicyandiamide as Co/N precursors for impregnation followed by annealing at 800 °C.^[^ ^26^ ^]^ However, in many cases, the Co‐loading level by wet impregnation method was relatively low (under 1 wt%), which limited the total number of the active sites for selective oxidations. Therefore, growing research of SACs is focusing on improving the doping level of Co single atoms. By selecting appropriate precursors and synthesis methods, the structure of the formed carbon support, the doped heteroatoms, and the interactions between Co and supports can be tuned to for enhanced catalytic performance. **Figure** **2**A–E lists some examples of SACo supported on nanocarbons from different Co, C, and N precursors. These processes were well designed and modified based on simple impregnation and pyrolysis methods to increase the doping level and stability of SACo. The formation of stabilized Co atoms starts with establishing strong Co–N association among the precursors. For instance, Co ions were successfully coordinated with the N atoms from 3,8‐diBr‐Phen (Figure 2A) or guanosine (Figure 2C). Cobalt (III) acetylacetonate can be coordinated with cyanamide (Figure 2B) and g‐C_3_N_4_ (Figure 2D). Some chemicals with natural Co—N bond can be directly used as precursor such as cobalt (II) phthalocyanine (Figure 2E). Then, precursors with chemically bonded Co and N underwent either polymerization or carbonization to generate SACo on N‐doped carbon, CNTs, and graphene. Specifically, for the synthesis of SACo@CNTs, excessive Co precursors were used as catalyst for the formation of CNTs. However, in many cases, the authors tried to use large amount of Co precursors to produce intermediate catalysts with both Co nanoparticles and single atoms where the particles were removed by acid leaching subsequently. This unavoidably caused waste of Co and involvement of corrosive acid. Liu and co‐workers managed to dope high loading of >3 wt% single Co atoms on g‐C_3_N_4_ and without the formation of nanoparticles by combining the impregnation and carbonization treatments. They first coordinated cobalt (II) phthalocyanine with citric acid and then impregnated the mixture with g‐C_3_N_4_. The solid was annealed at 655 °C to obtain the SACo@ g‐C_3_N_4_.

In addition to the aforementioned examples, some other Co–SACs were also developed with specific advantages. Xiong et al. reported the g‐C_3_N_4_ supported Co–SAC with an ultrahigh Co content of 23.58 wt% by pyrolysis of cobalt nitrate, formaldehyde, and dicyandiamide.^[^ ^27^ ^]^ Xia et al. incorporated S atoms into the carbon framework by introducing sulfuric acid into the pyrolysis precursors and elevated catalytic efficiency because the S doping facilitated the electron transfer on the surface of the SAC.^[^ ^25^ ^]^ Beyond that, Ji et al. reported the preparation of single Co atoms embedded in N/P co‐doped carbon from the pyrolysis of polymerized ZnCo–ZIF‐68, where the Zn atoms in the zeolitic imidazole frameworks (ZIFs) were eliminated during the high‐temperature annealing (950 °C) due to low boiling point of Zn.^[^ ^28^ ^]^ Co–SACs supported on other materials were reported where the supports and Co atoms made joint contributions to the target oxidation reactions. Co atoms encapsulated between MoS_2_ nanosheets were synthesized via electrochemical cointercalation for the oxidation of benzylic sulfides.^[^ ^29^ ^]^ The MoS_2_ nanosheets were dominant catalysts for the oxidation reaction while the incorporated Co atoms enhanced the adsorption of the reactants.

Overall, the development of Co–SAC preparation strategies for liquid‐phase selective oxidation system is complicated in the concerns of the concentration of SACo, stability, economic, and sustainability, and specific requirement form the target reaction. For carbon supports, g‐C_3_N_4_ contains natural structures of atom vacancies surrounded by N atoms, which is ready to accommodate the Co ions with higher loading level. Generally, the in situ pyrolysis methods with well‐stabilized Co–N chemical configuration can attain more Co single atoms than the simple wet impregnation treatments. The method conditions also have a great impact on the fabrication of SACs. For instance, a suitable annealing temperature is critical for both the formation of designed carbon morphology and to avoid the growth of Co particles at high temperatures.^[^ ^30^ ^]^ However, the development of facile, scalable, and low‐waste Co–SAC preparation routes to achieve high loading of Co atom is still lacking.

### 2.2 Characterization of Co–SACs

**Figure 3:**
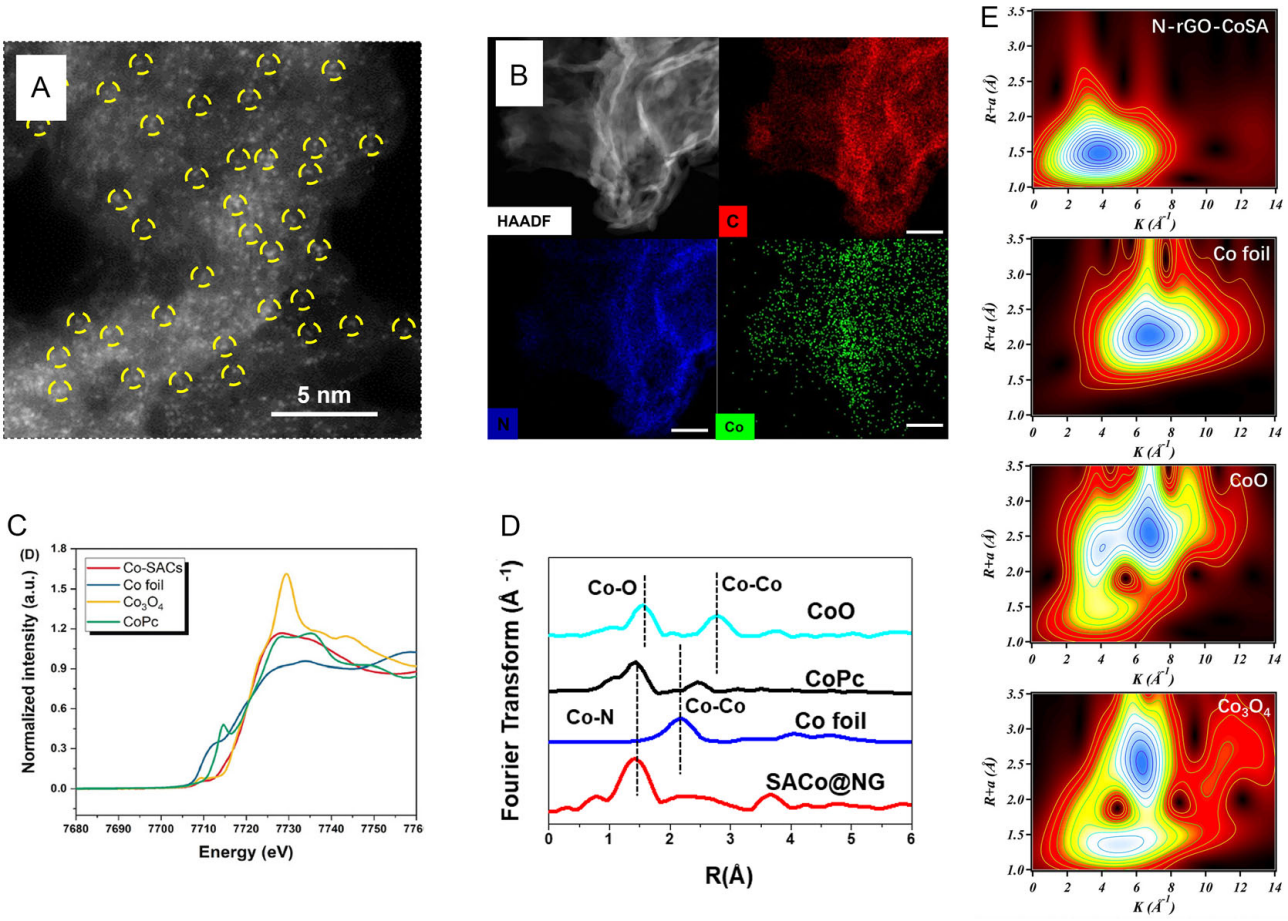
Representative characterizations of Co–SACs. A) high‐angle annular dark‐field scanning tunneling electron microscopy (HAADF–STEM) image of SACo@g‐C_3_N_4_. B) EDS mapping of the C/N/Co distribution of SACo@g‐C_3_N_4_. C) X‐ray absorption near‐edge structure (XANES), D) Fourier transforms of the extended X‐ray absorption fine structure (FT‐EXAFS), and E) wavelet transform of the EXAFS (WT‐EXAFS) of Co‐containing samples. Part (A,B) is reproduced with permission.^[^ ^31^ ^]^ Copyright 2021, The Royal Society of Chemistry. Part (C) is reproduced with permission.^[^ ^32^ ^]^ Copyright 2020, Wiley‐VCH Verlag GmbH & Co. KGaA, Weinheim. Part (D) is reproduced with permission.^[^ ^33^ ^]^ Copyright 2021, Wiley‐VCH GmbH. Part (E) is reproduced with permission.^[^ ^26^ ^]^ Copyright 2021, The American Chemical Society.

Two major characterization techniques are used as solid tools to confirm the atomic existence of Co and the chemical status of the Co atoms in SACs. One is the direct imaging of the Co atoms from high‐angle annular dark‐field scanning tunneling electron microscopy (HAADF–STEM). As shown in **Figure** **3**A, the single Co atoms on g‐C_3_N_4_ substrate can be recognized as bright dots because the heavier metal atoms are brighter than the background nonmetallic C/N/O atoms, meanwhile excluding the existence of larger‐sized nanoparticles. The energy dispersive spectrometry (EDS) elemental mapping (Figure 3B) confirms the presence and distribution of Co and N atoms on SACo@g‐C_3_N_4_.^[^ ^31^ ^]^ Nevertheless, HAADF–STEM image reflects regional appearance of Co atoms on SACs instead of the whole area statistical characterization. In this sense, extended X‐ray absorption fine structure (EXAFS) is used to identify the electronic structure and coordination environment of Co from the spectrum information. To be specific, the X‐ray absorption near edge structure (XANES) shows the behavior of Co species in absorption of photons and the Fourier transforms (FT) of the EXAFS monitors the bonding length of Co species. By employing the reference materials such as Co foil, Co oxides, and CoPc, the presence of the chemical bonds associated with Co atoms in Co—SAC can be confirmed. As can be seen from the XANES spectrum in Figure 3C, the absorption edge energy of Co—SAC at 7728.3 eV is close to that of Co phthalocyanine in which the Co were surrounded by four N atoms, suggesting that Co^2+^ with N coordination is highly possible structure in Co–SAC.^[^ ^32^ ^]^ FT‐EXAFS in Figure 3D exhibited a dominant peak of SACo@g‐C_3_N_4_ at 1.5 Å, the same as Co phthalocyanine, while far from the Co−O peak at 1.64 Å and the Co−Co peaks at 2.18 Å.^[^ ^33^ ^]^ The wavelet transform (WT) of the Co K‐edge EXAFS (Figure 3E) revealed the types of backscattering atoms of SACo@NG, which has one intensity maximum at 3.8 Å, different with the Co foil, CoO, and Co_3_O_4_.^[^ ^26^ ^]^ In addition, other characterizations such as X‐ray diffraction patterns (XRD), X‐ray photoelectron spectroscopy (XPS), and Fourier‐transform infrared spectra (FTIR) can also be applied to reflect the valence state of Co and exclude the presence of crystal Co structures. XRD patterns can be used to rule out the existence of crystal structured Co particles. XPS and FTIR spectra provide supporting evidence to the chemical valence states and the information of functionalities on the Co–SACs.

## 3 Model Oxidation Reactions for Co–SAC Evaluation

Co‐based SACs were applied to diverse liquid‐phase selective oxidation reactions. A typical model reaction to evaluate the catalytic performance is the selective oxidation of alcohols into aldehydes or ketones. Benzyl alcohol (BzOH) is used as a benchmark reactant because of its limited by‐products which greatly simplifies the reaction analysis process. The oxidation of benzylic alcohols is relatively easier than other substrates (e.g., hydrocarbons) to achieve close to 100% alcohol conversion and aldehyde/ketone selectivity, as the –OH groups are prone to adsorb on the N‐doped carbon materials, facilitating the surface activation of alcohols.^[^ ^34^ ^]^ Li and co‐authors reported the catalytic performance of SACo supported on MOF‐derived carbon (Co@P‐NC) in the selective oxidation of BzOH, achieving 99% conversion and excellent selectivity of 99% benzaldehyde.^[^ ^28^ ^]^ The activity (conversion) of Co@P‐NC was 10 times higher than Co nanoparticles on the same support and 50 times higher than Co^2+^ ion. Apart from the alcohol to aldehyde/ketone conversion, other oxidative reactions upon benzylic alcohols include the oxidative esterification of alcohols and the oxidative C—C bond cleavage from 1,2‐diols to obtain the corresponding esters, catalyzed by carbon‐supported Co–SACs.^[^ ^11^, ^35^ ^]^ Liu and co‐workers investigated the activity of SAM@NC (M = Co, Fe, Ni, and Cu) in the oxidative esterification of BzOH.^[^ ^11^ ^]^ Very surprisingly, the Co–SACs exhibited excellent activity to derive 99.8% conversion and 97.5% selectivity whereas the other three SACs only afforded ≈10% conversion with <1% selectivity at the same conditions. This highlights the superior catalytic performance of Co–SACs against the other transition metals toward some reactions.

**Figure 4:**
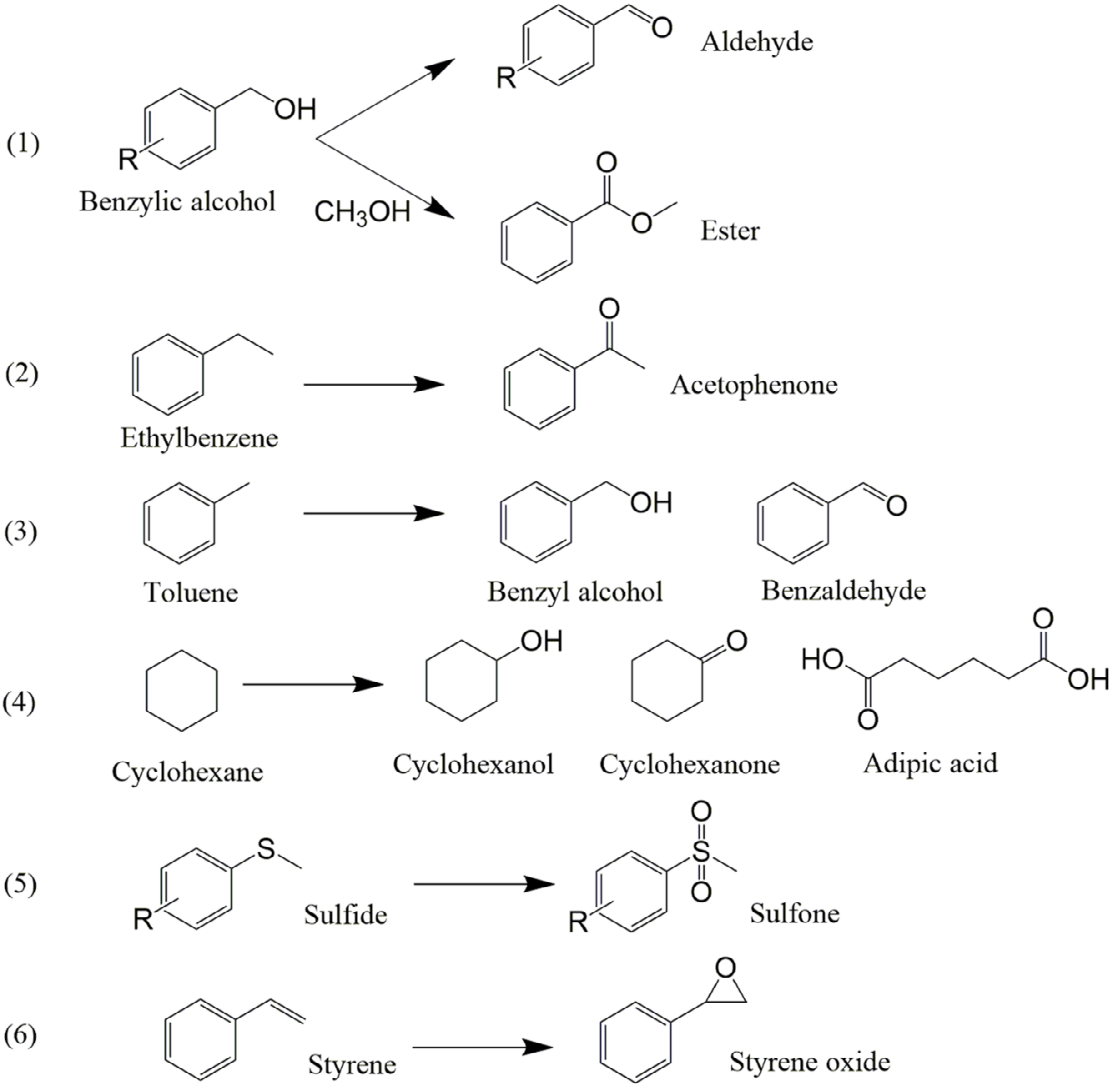
Currently applied selective oxidations over Co–SACs. 1) Selective oxidation and oxidative esterification of benzylic alcohol. 2–4) Oxidative C‐H bond activation of ethylbenzene, toluene and cyclohexane. 5) sulfide oxidation and 6) epoxidation of styrene.

Another extensively explored reaction in liquid‐phase selective oxidations is the C—H bond activation from hydrocarbons. The upgrading of hydrocarbons through oxidation treatments to derive oxygenated products is an essential task to extend the applications of hydrocarbons produced in petroleum industry. The oxidative conversion of ethylbenzene (EB) is a widely used model reaction of hydrocarbon activation. Although there are four possible products generated from EB oxidation, it was found that the activation of α‐C—H bond in EB is easier than that of the β‐C–H, leading to a high selectivity toward acetophenone (AP).^[^ ^36^ ^]^ Xia et al. reported EB oxidation by *tert*‐butylhydroperoxide (TBHP) over N‐graphene supported Co atoms. At a low temperature of 80 °C, the Co–SAC afforded 85% conversion and 95% selectivity toward ketone product, >2 times higher than the Co‐free carbon support.^[^ ^25^ ^]^ Xiong et al. investigated the EB oxidation in air where the Co SACs achieved product yield with a turnover frequency (TOF) of 19.6 h^−1^ whereas no reaction occurred on Co–porphyrin and Co NPs.^[^ ^27^ ^]^ The oxidations of other hydrocarbons including toluene and cyclohexane into the corresponding alcohol, aldehyde, ketone, and acid products were also studied over Co atoms deposited on CNTs and carbon nitride.^[^ ^17^, ^37^ ^]^ Beyond that, some other oxidative applications of Co–SACs in liquid‐phase reactions also include the epoxidation of styrene,^[^ ^38^ ^]^ sulfide oxidation,^[^ ^29^ ^]^ etc. The selective oxidation reactions currently reported with Co–SAC are summarized in **Figure** **4**.

Although Co–SACs presented great potential in liquid selective oxidations compared with nanocatalysts, their applications are still limited in model compounds and simple molecules. In the oxidative conversion of more complex substrates such as biomass polymers, the Co–SACs are still unexplored. We consider that Co–SACs possess potential in these industrial‐scale reactions. However, attention needs to be addressed to the low‐cost production of SACs with robust structures as the conversion of large molecule biomass requires harsh conditions.

## 4 Influence of the Type of Oxidants and Reaction Conditions

Traditionally, high‐valence metal‐based oxidants such as dichromate (Cr_2_O_7_^2−^) and permanganate (MnO_4_^−^) are employed for both lab‐scale and industrial synthesis of fine chemicals.^[^ ^39^ ^]^ Despite the high oxidation efficiency, a large amount of toxic and corrosive waste is produced in these conventional processes, which is not competitive in terms of seeking environmental sustainability. Therefore, many green oxidants have been developed for sustainable selective oxidation processes. The major oxidants associated with Co–SACs in selective oxidation processes are divided into two fundamental classes: oxygen gas/air and a series of liquid/solid peroxide oxidants. From the viewpoint of environment and economic benefits, using pure O_2_ gas or directly using air to perform selective oxidation is ideal to achieve a cost‐effective and green chemical process. Tremendous research efforts have been devoted to O_2_ utilization toward liquid‐phase selective oxidation reactions, but high energy is required for the activation of O_2_ due to the inertness of ground‐state O_2_. In this sense, the highly efficient selective oxidation from O_2_ is still a challenging task, especially for the stubborn C—H bond activation in hydrocarbon oxidations. To obtain satisfying reaction yield, relatively high reaction temperature, high O_2_ pressure, cocatalysts, or additives were frequently adopted. Li et al. reported the selective oxidation of BzOH in pure O_2_ at 130 °C over Co single atoms supported on N‐doped graphene (NG) to gain 92.4% benzaldehyde (BzH) yield after 5 h reaction.^[^ ^40^ ^]^ The solvent‐free oxidation of EB in air atmosphere at 120 °C for 24 h catalyzed by Co–SAC on carbon nitride was performed by Li and co‐workers.^[^ ^27^ ^]^ An EB conversion of 46%, AP selectivity of 97%, and a high TOF of 19.6 h^−1^ were derived from their research. Toluene oxidation catalyzed by Co@CNTs showed low conversion (28%) even at high temperature (140 °C) and pressure (3 MPa O_2_) due to the stubbornness of toluene molecules.^[^ ^37^ ^]^

Peroxide oxidants also made great contributions to the studies of selective oxidation reactions. Hydrogen peroxide was used for the conversion of benzylic primary alcohols into aldehydes catalyzed by Co atoms stabilized on N and P co‐doped carbon.^[^ ^28^ ^]^ More than 99% alcohol conversion and aldehyde selectivity were obtained over most primary alcohols at 110 °C, indicating that H_2_O_2_ exhibited higher oxidation efficiency as it is easier to be activated to generate oxidizing species relative to O_2_. TBHP has been widely used in EB oxidation with Co–SACs and showed higher reactivity than H_2_O_2_ owing to a weaker O—O bond that requires lower activation energy. As a result, the combined TBHP/Co–SAC system can afford at least 90% EB conversion at temperatures lower than 80 °C.^[^ ^41^ ^]^ In particular, 98% EB conversion and 99% AP selectivity were achieved at room temperature catalyzed by Co single atoms anchored on porous carbon as reported by Li and co‐workers.^[^ ^36^ ^]^

In addition to the gas‐phase oxygen and liquid peroxides, Co–SACs have also been reported to activate environmentally benign solid salt oxidants, including peroxymonosulfate (PMS, HSO_5_^−^) peroxydisulfate (PDS, S_2_O_8_^2−^),^[^ ^42^ ^]^ and periodate (IO_4_^−^).^[^ ^26^ ^]^ Compared with gas and liquid oxidants, these solid oxidants are more stable for transportation and storage and have limited impact on the environment. The PMS molecules with asymmetric structure are more easily to be activated than PDS to generate oxidizing species. However, the activation of PMS/PDS/IO_4_^−^ was commonly applied to advanced oxidation processes (AOPs) to fully degrade the organic pollutants into inorganic CO_2_ and H_2_O,^[^ ^43^ ^]^ but they were rarely used for selective oxidation reactions to controllably synthesize the oxygenated organics.^[^ ^44^ ^]^ Recently, Liu and co‐workers synthesized Co–SACs on carbon substrates (graphene and g‐C_3_N_4_) to activate PMS for highly efficient selective oxidation of BzOH and EB.^[^ ^31^, ^33^ ^]^ The reactions were performed at temperatures below 60 °C to gain over 90% yield of the target product, successfully expended the application of persulfate salts toward liquid‐phase selective oxidations. A comparison of the properties and performances of different oxidants in selective oxidation applications is displayed in **Table** **1**.

**Table 1:** Comparison of the properties of different oxidants in selective oxidation applications

Properties	O_2_/air	H_2_O_2_/TBHP	Solid salts
Production cost and availability	Cheap and abundant	Medium cost and availability	Medium cost and availability
Activation energy	High	Medium/low	Low
Environmental impact	Green and clean	Clean/minor impact to environment	Minor impact to environment
Storage and transportation stability	Inconvenient for transportation and storage	Unstable for long‐term storage and not safe for transportation	More stable and safer than liquid and gas for transportation and storage
Research progress	Received intensive research interest from environmental concerns	Widely used as highly efficient oxidant	Mainly applied for AOPs and newly used in selective oxidations

Although peroxide oxidants can afford higher activity than O_2_, their influence on the stability of Co–SACs was also greater because the oxidative/acidic reaction environment may cause the Co leaching to reduce the amount of Co atoms and raise environmental concerns. In addition, the partial aggregation of SACo during the reaction at high temperature may also lead to reduced catalytic activity. Overall, the stability of the carbon‐supported Co–SACs is satisfying with less than 10% decrease of oxidation efficiency after 4–5 reaction cycles in most of the oxidation reactions. To further improve the stability, the chemical bonding between Co atoms and the supporting substrates needs to be strengthened by special treatment during the preparation. The following solution are suggested to achieve this: 1) using periodic on–off heating (55 ms on and 550 ms off) at very high temperatures can promote the bond between single atoms and the supports;^[^ ^45^ ^]^ 2) for SACs embedded on metal‐based supports, high‐temperature phase change of the metal supports can create oxygen vacancies to strongly anchor the single‐atom sites;^[^ ^46^ ^]^ 3) spatial confinement of the single atoms supports with porous structure or abundant vacancies such as zeolites and g‐C_3_N_4_.

## 5 Mechanism Study of Oxidant Activation and Selective Oxidation

**Figure 5:**
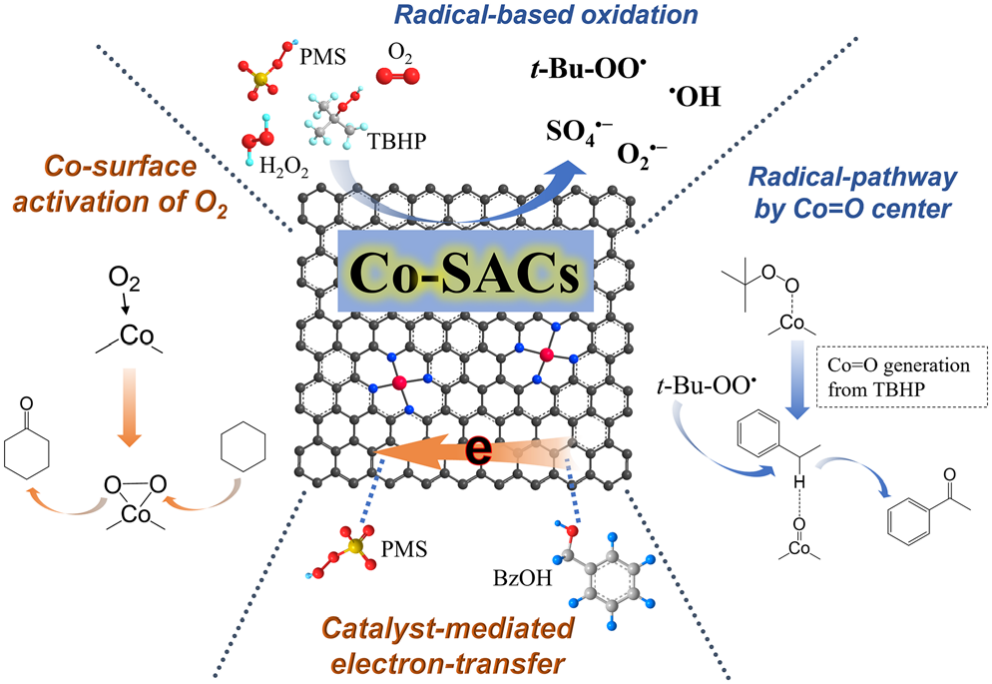
Mechanisms proposed for oxidant activation and selective oxidation over Co–SACs.

The scope of mechanism study for Co–SAC‐catalyzed selective oxidation includes the identification of catalytic active sites, the activation process of the oxidants, and the reaction pathways of the oxidation of organic substrates. The catalytic activity of Co–SACs was dominantly attributed to the Co–N_*x*_ species, confirmed by adding thiocyanate (SCN^−^) as a Co‐poisoning agent which could selectively react with the Co–N_*x*_ to form a metal complex.^[^ ^25^, ^37^ ^]^ But the catalytic role of the carbon supporting material and nitrogen species received diverse conclusions over different reaction systems. For instance, the N‐doped carbon system was found inactive for toluene oxidation by O_2_,^[^ ^37^ ^]^ but it contributed a small activity in EB oxidation from TBHP and a substantial activity in PMS activation.^[^ ^33^ ^]^ In this sense, different oxidants react distinctively toward the Co–SAC during activation owing to their inherent chemical structures. The reaction mechanism of selective oxidation processes is generally classified into radical processes and non‐radical processes. The selective oxidations were dominantly reported to occur via radical pathways, with different radicals formed from electron gain or loss during the activation of oxidants, including superoxide radical anions (^•^O_2_^−^) from O_2_ activation,^[^ ^37^ ^]^ *tert*‐butyloxygen radicals (*t‐*BuO^•^) from TBHP,^[^ ^36^ ^]^ hydroxyl radicals (^•^OH) from H_2_O_2_,^[^ ^29^ ^]^ and sulfate radicals (SO_4_^•−^) from PMS/PS.^[^ ^31^ ^]^ Chen et al. suggested that the Co–N_4_ configuration of Co–SAC can turn the surface adsorbed O_2_ into ^•^O_2_^−^ by electron transfer for subsequent radical‐based conversion of toluene into benzoic acid.^[^ ^37^ ^]^ Zhu et al. reported that both *t‐*BuO^•^ and ^•^OH radicals can be produced from TBHP catalyzed by the Co‐active centers to subsequently attack the C—H bond in EB.^[^ ^36^ ^]^ They also indicated that the Co=O centers generated from TBHP oxidation played an important role for radical‐based EB oxidation. In contrast with the radical process, some selective oxidation systems over Co–SACs were discovered to be accomplished through nonradical routes without generating free radicals. Hou and co‐workers proposed that the cyclohexane can be oxidized by the O_2_ molecules adsorbed/activated on Co single atoms of SACo@g‐C_3_N_4_ to form a reactive complex without involving the radical chain reactions.^[^ ^17^ ^]^ Li et al. reported the nonradical oxidation of BzOH by activated PMS through the surface adsorption of PMS and BzOH on SACo@NG catalyst and the direct electron transfer from BzOH to PMS via the graphene framework.^[^ ^33^ ^]^ They also confirmed that the non‐radical pathway is milder and more selective than the highly oxidative radicals to yield higher product selectivity.^[^ ^34^ ^]^ A schematic summarization of the reaction mechanisms over Co–SACs in selective oxidation is displayed in **Figure** **5**.

Many strategies have been developed to explore the mechanisms by experimental or computational studies. Several radical quenching agents were used for experimental verification of the radical pathways, including p‐benzoquinone, hydroquinone, isopropanol, 2,2,6,6‐tetramethyl‐1‐piperidinyloxy, acrylamide, and butylated hydroxytoluene.^[^ ^17^, ^29^, ^35^, ^37^ ^]^ However, the accuracy of the radical quenching method to determine the role of each radical is limited because the scavenging properties of the quenching agents may overlap and are lack of absolute specificity. In addition to the radical quenching treatments, the presence of radicals during the catalytic activation of oxidants could also be monitored by in situ electron paramagnetic resonance using 5,5‐dimethyl‐1‐pyrroline N‐oxide to capture the free radicals.^[^ ^11^ ^]^ Different radical species can be recognized from the patterns of the peak signals.

**Figure 6:**
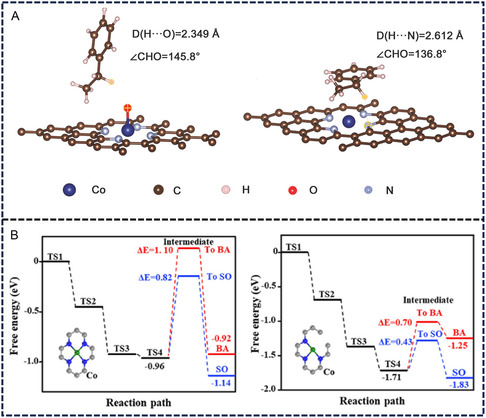
A) Adsorption of ethylbenzene on N–Co=O and N–Co configurations. B) Free energy diagrams on Co–N_4_ and Co–N_3_ structures for oxidation of styrene. Part (A) is reproduced with permission.^[^ ^36^ ^]^ Copyright 2018, WILEY‐VCH Verlag GmbH & Co. KGaA, Weinheim. Part (B) is reproduced with permission.^[^ ^38^ ^]^ Copyright 2022, The American Chemical Society.

Moreover, density‐functional theory (DFT) calculations were frequently performed to theoretically analyze the thermodynamic properties, electronic structures, and interactions among the catalysts, oxidants, and organic substrates. First, the adsorption energy of the oxidant and organic molecules upon the catalysts can be calculated to confirm the active sites as the surface adsorption is a prerequisite step for the catalytic activation of the reactants. As shown in **Figure** **6**A, Co=O configuration was detected during the selective oxidation of EB by TBHP on SACo@NC catalyst. Zhu et al. calculated the adsorption behavior of C—H bond in EB molecules on Co=O and Co—N, respectively, and the results suggested a shorter bond distance of C—H…O compared with that of C—H…N. Thus, the Co=O structure is more favorable for C—H bond cleavage.^[^ ^36^ ^]^ Second, DFT calculations can provide the most possible pathway for the selective oxidation reactions with the catalytic activated oxidants by comparing the energy barriers for the pathways from different intermediates.^[^ ^38^ ^]^ Third, the calculation results can also predict the optimal structure of the catalytic active sites, which can provide guidance for the synthesis of efficient SACs. For example, Jia et al. calculated the energy barrier of the oxidation/epoxidation of styrene to benzaldehyde and styrene oxide on Co–N_4_ and Co–N_3_ structures. It was found that the energy barrier for the product formation on unsaturated Co–N_3_ structures (0.43/0.70 eV) is much lower than those on Co–N_4_ (0.82/1.10 eV) (Figure 6B).^[^ ^38^ ^]^ Therefore, the Co–N_3_ structure is more desirable for the catalytic oxidation of styrene.

One challenge of the mechanism study in Co–SACs catalyzed reaction is the carbon support. Unlike crystal materials, carbon can form various materials with diverse morphology and structures. Even for highly ordered graphene and CNTs, there are heteroatoms, functional groups, and defects which lead to uncertainties for the identification of active sites and reaction mechanism. Current DFT research for mechanism study on carbon‐supported Co–SAC normally focused on optimized geometries with Co–N configuration while ignoring the potential interaction of Co with other functionalities and structural defects, etc. Another supportive strategy for mechanism study is in situ technology. In situ Raman, in situ infrared spectroscopy, and in situ X‐ray absorption spectroscopy (XAS) study can probe the evolution of the surface structure and composition of the catalyst during the reaction. However, such techniques were rarely used for Co–SACs in liquid oxidation systems. Further research can be performed regarding these concerns.

## 6 Summary

Cobalt‐based SACs have been recognized as highly efficient catalysts in liquid‐phase selective oxidation processes, combining the merits of both heterogeneous and homogeneous catalysis. Co possesses advanced catalytic activity among the transition metals in many oxidative reactions, showing great potential as a substitute for noble‐metal catalysts in synthetic chemistry. Currently, the chemically stabilized Co atoms through the Co–N_*x*_ configuration on carbon‐based supports is the most widely studied form of Co–SACs. To increase the exposed single Co atom sites, numerous efforts are applied to both in situ synthesis and post‐preparation methods for the fabrication of SACs with high loading of single Co atoms. There are diverse doping levels of atomic Co in reported research by adjusting the Co/C/N precursors and the additives and through process engineering. The concentration of the Co atoms in SACs significantly influenced the catalytic efficiency. Yuan et al. monitored the cyclohexane oxidation catalyzed by a series of SACo@g‐C_3_N_4_ catalysts with varying Co contents between 0.03% and 0.09%.^[^ ^17^ ^]^ The reaction conversion remarkably increased with higher SACo loading. However, the facile and scalable strategies to prepare Co–SACs are still highly desired. The produced Co–SACs were successfully used in the selective oxidations of a broad range of organics, e.g., primary alcohols, aromatic hydrocarbons, sulfides, and styrene, but the application in the conversion of more complex substrates such as biomass requires further attention in the scope of industrial‐scale utilization. Different types of oxidants have been adopted to enable the selective oxidations catalyzed by Co–SACs, where the liquid and solid peroxides display superior oxidation efficiency toward gaseous oxygen, but the stability and cyclability of the Co–SACs need to be improved to prevent the Co‐ion leaching and aggregation. In this regard, strong chemical bonds between the Co atoms and the supporting materials should be attained against the oxidative/acidic reaction environments. The possible strategies to achieve this target are suggested in this review. Both experimental and computational efforts are devoted to the mechanism research to provide deeper insights into the intrinsic active sites, and to clarify the activation of oxidants by Co–SACs and the evolution of organic substrates during the oxidation process. Radical‐based process still dominates the selective oxidation, while the less reported nonradical‐based oxidation may deliver a more controllable and selective way, which deserves more attention in the future. For mechanism exploration via experimental study, more detailed study of the interaction between Co atoms and the carbon support is suggested. In situ techniques such as in situ XAS, Raman, and IR needed to be performed to probe the reaction process. Based on the high potential of Co–SACs in this field, more research is suggested for both the development of novel catalysts and their broader applications in liquid‐phase selective oxidation to gain a comprehensive understanding of the catalytic performance, which will no doubt build foundations to expand their practical applications.

## Conflict of Interest

The authors declare no conflict of interest.
